# Emerging role of 18F-FDG PET/CT in Castleman disease: a review

**DOI:** 10.1186/s13244-021-00963-1

**Published:** 2021-03-11

**Authors:** Benjamin Koa, Austin J. Borja, Mahmoud Aly, Sayuri Padmanabhan, Joseph Tran, Vincent Zhang, Chaitanya Rojulpote, Sheila K. Pierson, Mark-Avery Tamakloe, Johnson S. Khor, Thomas J. Werner, David C. Fajgenbaum, Abass Alavi, Mona-Elisabeth Revheim

**Affiliations:** 1 Department of Radiology, University of Pennsylvania, Philadelphia, PA USA; 2 Drexel University College of Medicine, Philadelphia, PA USA; 3 Perelman School of Medicine at the University of Pennsylvania, Philadelphia, PA USA; 4 Department of Medicine, Division of Translational Medicine and Human Genetics, University of Pennsylvania, Philadelphia, PA USA; 5 Division of Radiology and Nuclear Medicine, Oslo University Hospital, Sognsvannsveien 20, 0372 Oslo, Norway; 6 Institute of Clinical Medicine, Faculty of Medicine, University of Oslo, Problemveien 7, 0316 Oslo, Norway

**Keywords:** Castleman disease, Positron emission tomography/computed tomography, Fluorodeoxyglucose F18, Interleukin-6, HIV

## Abstract

Castleman disease (CD) describes a group of rare hematologic conditions involving lymphadenopathy with characteristic histopathology and a spectrum of clinical abnormalities. CD is divided into localized or unicentric CD (UCD) and multicentric CD (MCD) by imaging. MCD is further divided based on etiological driver into human herpesvirus-8-associated MCD, POEMS-associated MCD, and idiopathic MCD. There is notable heterogeneity across MCD, but increased level of pro-inflammatory cytokines, particularly interleukin-6, is an established disease driver in a portion of patients. FDG-PET/CT can help determine UCD versus MCD, evaluate for neoplastic conditions that can mimic MCD clinico-pathologically, and monitor therapy responses. CD requires more robust characterization, earlier diagnosis, and an accurate tool for both monitoring and treatment response evaluation; FDG-PET/CT is particularly suited for this. Moving forward, future prospective studies should further characterize the use of FDG-PET/CT in CD and specifically explore the utility of global disease assessment and dual time point imaging.

*Trial registration* ClinicalTrials.gov, NCT02817997, Registered 29 June 2016, https://clinicaltrials.gov/ct2/show/NCT02817997

## Key Points

- Castleman disease (CD) describes a group of rare hematologic disorders involving lymphadenopathy with characteristic histopathology and a spectrum of clinical abnormalities.
- FDG-PET/CT allows detection of inflammation at a molecular level in CD that may proceed structural changes detected by CT and MRI.
- FDG-PET/CT can contribute to correct sub-classification of CD into unicentric CD (UCD) and multicentric CD (MCD).

## Background

Castleman disease (CD), also known as angiofollicular lymph node hyperplasia and giant lymph node hyperplasia, is a hematologic disorder first reported by Benjamin Castleman in 1954. CD is a rare disease diagnosed in 6600–7700 individuals each year in the USA [1]. No data suggest a strong gender predilection [2]. All CD patients are present with lymphadenopathy that demonstrates characteristic histopathological changes and a spectrum of clinical abnormalities.

**Fig. 1:**
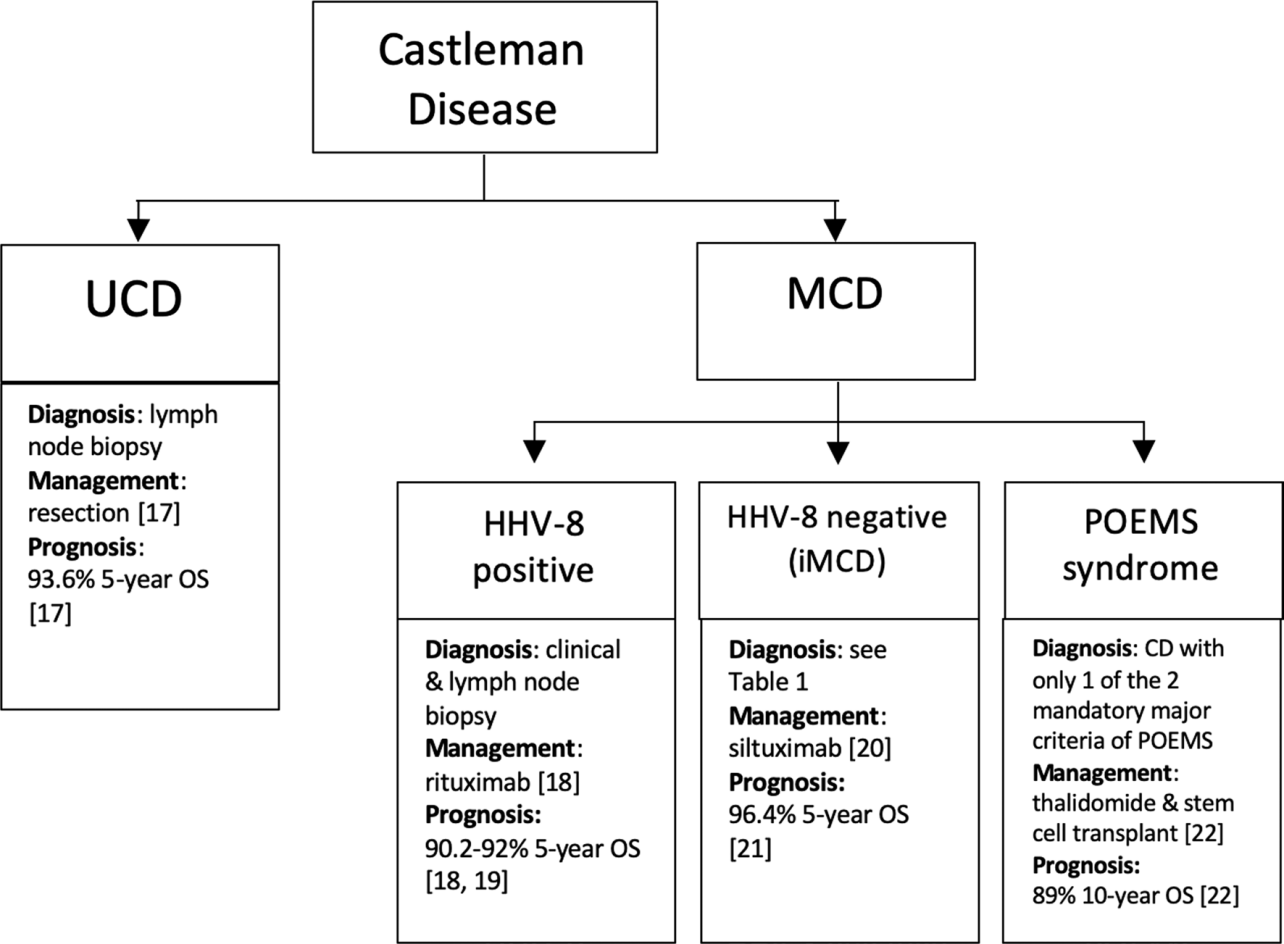
Summary of the main types of Castleman disease [17–22]. UCD, unicentric Castleman disease; MCD, multicentric Castleman disease; HHV-8, human herpesvirus-8; iMCD, idiopathic multicentric Castleman disease; POEMS, polyneuropathy, organomegaly, endocrinopathy, monoclonal protein, skin changes

CD is sub-classified based on the number of enlarged lymph nodes. Unicentric CD (UCD) involves a single lymph node or a single region of nodes, while multicentric CD (MCD) involves multiple lymphatic stations [3]. Available data suggest that UCD is more common than MCD [1]. UCD has been reported to occur in younger individuals than MCD, though epidemiologic data are sparse, and both can occur in individuals of all ages [4, 5]. Most cases of UCD are located in the mediastinum, but UCD can involve any lymph node region in the body [6–9]. In contrast, MCD involves lymphadenopathy in greater than one region and can occur in any region of the body. Additionally, MCD has a poorer prognosis than UCD. MCD is further divided into three subgroups: human herpesvirus 8 (HHV-8)-associated MCD; polyneuropathy, organomegaly, endocrinopathy, monoclonal gammopathy, skin changes (POEMS)-associated MCD; and idiopathic MCD (iMCD) [10]. The relative breakdown between HHV-8-associated MCD, POEMS-associated MCD, and iMCD is not well characterized. These four groups vary considerably in clinical characteristics, preferred treatment, and clinical outcomes. Therefore, correct classification is vital at the time of diagnosis. In addition, many diseases are present with CD-like clinicopathology, including lymphoma, Kaposi sarcoma, immunoglobulin G4-related disease (IgG4-RD), and other benign and malignant tumors [11–14]. Furthermore, HHV-8-associated MCD is often seen in human immunodeficiency virus (HIV)-positive or otherwise immunosuppressed patients who are at increased risk for such CD-like diseases [4, 15, 16]. As such, the exclusion of pathologies that can mimic CD is critical. The features, prognosis, and management of each main subtypes of CD are summarized in Fig. 1 [17–22].

The standard investigative workup in Castleman disease usually begins with lymph node biopsy followed by radiological investigation with PET/CT preferred, complete blood count, serum chemistry, markers of inflammation, serum cytokine levels, viral serology for HHV-8 and HIV, and protein electrophoresis, immunofixation, and quantitative immunoglobulins [23]. To further exclude other similarly presenting conditions, clinical findings must be considered and other laboratories can be ordered such as IgH gene arrangement study on the biopsied lymph node to rule out lymphoma and serology investigations for autoimmune disorders [23]. A formal diagnostic criteria have only been established for iMCD and are summarized in Table 1 [4]. Diagnosis of HHV-8 associated MCD generally requires HHV-8 detection, lymphadenopathy in multiple regions, and plasmablastic histopathologic findings on lymph node biopsy [24]. POEMS-associated MCD is diagnosed if only one of two the mandatory major criteria of polyneuropathy and monoclonal plasma proliferative disorder needed for diagnosis of POEMS syndrome is present with lymph node biopsy diagnostic of CD [25].

**Table 1:** From the iMCD consensus diagnostic criteria proposed from The International Working Group for iMCD [4]

**Major criteria** 1. Lymph nodes with histopathologic features consistent with iMCD spectrum 2. Enlarged lymph nodes (≥ 1 cm in short-axis diameter) in ≥ 2 lymph node regions
**Minor criteria** Laboratory 1. Elevated CRP or ESR 2. Anemia 3. Thrombocytopenia or thrombocytosis 4. Hypoalbuminemia 5. Renal dysfunction or proteinuria 6. Polyclonal hypergammaglobulinemia **Clinical** 1. B symptoms 2. Hepatomegaly or splenomegaly 3. Fluid accumulation 4. Eruptive cherry hemangiomatosis 5. Violaceous papules 6. Lymphocytic interstitial pneumonitis
**Supporting features** 1. Elevated IL-6, VEGF, IgA, IgE, LDH, and/or B2M 2. Reticulin fibrosis of bone marrow 3. Disorders associated with iMCD: paraneoplastic pemphigus, bronchiolitis obliterans organizing pneumonia, autoimmune cytopenias, polyneuropathy, inflammatory myofibroblastic tumor
**Exclusion criteria** 1. Infection: HHV-8, EBV, CMV, toxoplasmosis, HIV, active tuberculosis 2. Autoimmune/autoinflammatory: systemic lupus erythematosus, rheumatoid arthritis, adult-onset Still disease, juvenile idiopathic arthritis, autoimmune lymphoproliferative syndrome 3. Malignancy: lymphoma, multiple myeloma, POEMS syndrome, primary lymph node plasmacytoma, follicular dendritic cell sarcoma

^*^Diagnosis requires both major criteria and at least 2 of 11 minor criteria with 1 laboratory criterion. Diseases that can mimic iMCD listed in the exclusion criteria must be ruled out

iMCD, idiopathic Castleman disease; CRP, C-reactive protein; ESR, erythrocyte sedimentation rate; IL-6, interleukin 6; VEGF, vascular endothelial growth factor; IgA, immunoglobulin A; IgE, immunoglobulin E; LDH, lactate dehydrogenase; B2M, Beta-2 microglobulin; HHV-8, human herpesvirus-8; EBV, Epstein–Barr virus; CMV, cytomegalovirus; POEMS, polyneuropathy, organomegaly, endocrinopathy, monoclonal protein, skin changes)

Excessive cytokine production is believed to underlie CD pathogenesis. UCD and POEMS-associated MCD are believed to be caused by somatic mutations in monoclonal stromal and plasma cells, respectively, resulting in excessive cytokine release and subsequent clinical symptomatology [26]. In HHV-8-associated MCD, uncontrolled infection with HHV-8 occurs due to host immunocompromise, leads to a viral interleukin (IL)-6 driven cytokine storm, and correlates with symptom severity [27–30]. Finally, while the precise mechanism of iMCD is not known, elevated IL-6 associated with autoimmune mechanisms, ectopic cytokine secretion by tumor cells, and/or viral signaling by a non-HHV-8 virus have been proposed as possible etiologies [15]. IL-6 levels directly parallel disease activity, and elevated serum IL-6 induces B-lymphocyte growth, secretion of vascular endothelial growth factor (VEGF), and inflammatory responses. Also, mice with retroviral transduction of IL-6 coding sequence within hematopoietic stem cells develop an MCD-like syndrome [31]. As we discuss in the following sections, the modulation of IL-6 and other inflammatory cytokines is effective in a large portion of iMCD patients.

## Common presenting symptoms and laboratory abnormalities in CD

UCD may be clinically silent, and recent evidence suggests that most UCD patients do not demonstrate any signs or symptoms beyond solitary lymphadenopathy [32]. That being said, UCD may predispose patients to malignancy [33, 34]. Laboratory findings, including complete blood count and inflammatory markers, are typically unremarkable in UCD patients.

In contrast, the three subtypes of MCD (HHV-8-associated MCD, POEMS-associated MCD, iMCD) present with diffuse lymphadenopathy, systemic inflammation, and organ dysfunction. These patients typically demonstrate B symptoms (fever, chills, night sweats), cough, thoracic or abdominal pain, dyspnea, weight loss, and hemoptysis [32]. Comorbid malignancies, including lymphoma in iMCD and Kaposi’s sarcoma in HHV-8-associated MCD, have been found to occur [31, 33, 34]. In addition, patients with the three subtypes of MCD may demonstrate numerous laboratory abnormalities, including anemia, leukocytosis, thrombocytopenia and thrombocytosis, elevated inflammatory markers (C-reactive protein, IL-6, and erythrocyte sedimentation rates), elevated IgG, hypoalbuminemia, renal dysfunction, and elevated liver enzymes [1, 31]. Of note, serological tests for hepatitis B virus, cryoglobulin, antinuclear antibody, and cytomegalovirus are usually negative [35]. Consensus diagnostic criteria exist for iMCD and for POEMS syndrome, which should both be evaluated in all potential MCD cases. The heterogeneity of CD presents a challenge, so both clinical and laboratory findings must be carefully considered in the diagnosis and workup for suspected CD [4, 25].

## Role of FDG-PET/CT in the diagnosis of CD

18F-fluorodeoxyglucose-positron emission tomography/computed tomography (FDG-PET/CT) can be used to assess the metabolic activity of the enlarged lymph nodes in CD. FDG is a radiolabeled glucose analog taken up preferentially by metabolically active cells, such as malignant tumor cells or inflammatory cells [9]. Currently, FDG-PET/CT is recommended as an alternative to CT scan alone in the published iMCD treatment guidelines [36]. However, the potential for FDG-PET/CT in the diagnosis, treatment assessment, and follow-up of CD has not been fully demonstrated. With the ability to collect structural and metabolic information, FDG-PET/CT can enhance the specificity and sensitivity in identifying affected lymph nodes in CD patients [11]. Specifically, contrast-enhanced PET/CT would provide joint functionality of both contrast-enhanced CT and PET.

Currently, CT is routinely utilized in CD patients to identify and characterize lymph nodes by size, shape, and contrast enhancement pattern [7]. The lymphadenopathy in UCD, HHV-8-associated MCD, POEMS-associated MCD, and iMCD typically demonstrates marked and rapid contrast enhancement on CT [37]. HHV-8-associated MCD, POEMS-associated MCD, and iMCD may additionally present with hepatosplenomegaly and other organ-specific imaging anomalies. Of note, thickening of the lung septa, bronchovascular bundles, and centrilobular nodules may present on CT as thin-walled cysts and ground-glass opacity [8, 9, 13].

A major limitation of CT is that it cannot sensitively detect the involvement of normal-sized lymph nodes, nor can it distinguish between reactive hyperplasia and pathological enlargement of lymph nodes. Additionally, CD can sometimes be misinterpreted via CT and magnetic resonance imaging (MRI) as other similarly appearing autoimmune, malignant, or infectious disorders [3, 4, 11, 14] (Table 2). For example, thoracic UCD may be interpreted as a thyroid mass, parathyroid adenoma, or hemangiomas [9].

**Table 2:** Autoimmune, neoplastic, and infectious disorders with significant shared clinical, histologic, and immunologic features of Castleman disease (CD) [3, 4, 11, 14]

Types of disorders	Disorders
Autoimmune	Immunoglobulin G4-related disease, rheumatoid arthritis (RA), systemic lupus erythematosus (SLE), adult-onset Still Disease
Neoplastic	Lymphoma, desmoid tumors, retroperitoneal sarcoma, paragangliomas, sarcomas, hemangiopericytoma, bronchial adenoma, neurofibroma, chest wall tumors, schwannoma
Infectious	Human immunodeficiency Virus (HIV), Epstein–Barr virus (EBV), cytomegalovirus, tuberculosis, toxoplasmosis

Fused FDG-PET/CT takes into account the metabolic characteristics of the structures [38]. This molecular imaging modality, thus, can detect abnormally high uptake in small lymph nodes that would be overlooked by purely structural imaging modalities and facilitate a correct and complete diagnosis. Also, FDG-PET/CT can identify lymph nodes and lesions that are more likely to yield a definitive diagnosis on biopsy [39]. Even in cases of UCD with only mild contrast enhancement on CT, focal lymphadenopathy can demonstrate increased FDG uptake. The majority of HHV-8-associated MCD patients also show increased FDG uptake in the bone marrow and the spleen [39]. In addition, lung parenchymal changes in iMCD may be confirmed by increased FDG uptake. That being said, many institutions may not implement breath-holds for the pulmonary portion of the scans, potentially obscuring fine details of the lung parenchyma and leading to less accurate longitudinal measurement of small lung lesions due to respiratory motion artifact [40, 41]. In these cases, additional techniques to correct for respiratory motion artifacts, such as phase-aligned correction and gating algorithms, are warranted [42, 43].

The degree of FDG uptake may be different between UCD, HHV-8-associated MCD, POEMS-associated MCD, and iMCD. While one study reported a significantly higher lymph nodes maximum standardized uptake value (SUVmax) in MCD versus UCD, another study noted no significant difference [44, 45]. Clinical manifestations of CD have also been shown to be correlated with the degree of FDG uptake in a small study [38]. However, the use of SUV as the sole means of differentiation between subtypes has significant limitations. SUV is specific to the technique and instrumentation used, which is highly variable among institutions [46, 47]. Nevertheless, PET/CT scanners and the imaging protocol adhered are increasingly standardized to international practice guidelines and the PET/CT systems including calibration and harmonized to a phantom reduce the variation of quantitation parameters [48, 49].

Although some evidence indicates that FDG-PET/CT may be utilized to differentiate between CD subtypes, as well as between CD and lymphoma as potential causes of lymphadenopathy, additional studies must be performed to corroborate these findings [34, 45, 50–52]. In addition, MCD patients are at increased risk for the development of lymphoma, which can confound FDG uptake findings [53]. A potential confounder for the use of FDG in the evaluation of lymphadenopathy is sarcoidosis, which can resemble lymphoma and CD both morphologically and metabolically, and may rarely coexist with CD [54]. Laboratory findings may help differentiate other inflammatory conditions from CD; however, histopathological evaluation through tissue biopsy is the recommended approach in evaluating unexplained lymphadenopathy [55].

Although excisional lymph node biopsy is the only way to definitively diagnose CD based on its histology, existing evidence suggests that FDG-PET/CT should be performed beforehand to help determine CD subtype, consider the possibility of lymphoma, and identify ideal lesions for biopsy [39, 45].

## FDG-PET/CT in treatment assessment and monitoring of CD

Treatment options for CD and responses differ by subtype. For UCD, complete surgical resection of the node is an effective and usually curative treatment. When complete surgical excision cannot be performed, chemotherapy and radiation therapy are alternative therapies that sometimes can be followed by resection [56, 57].

The first-line treatment for HHV-8-associated MCD involves rituximab, an anti-CD20 monoclonal antibody, with or without antivirals and liposomal doxorubicin. Treatment of POEMS-associated MCD is directed against the underlying POEMS syndrome using immunomodulators and autologous stem cell transplantation. iMCD is treated first-line with the anti-IL-6 immunotherapy siltuximab (the only FDA-approved treatment for iMCD), which may be combined with corticosteroids. iMCD refractory to siltuximab may be treated with rituximab, immunosuppressants (e.g., sirolimus, cyclosporine), immunomodulators (e.g., thalidomide, bortezomib), or multi-agent cytotoxic chemotherapy for severe disease [58]. With more insight into disease mechanisms and signal pathways involved in iMCD, new treatment strategies are under investigation.

**Fig. 2:**
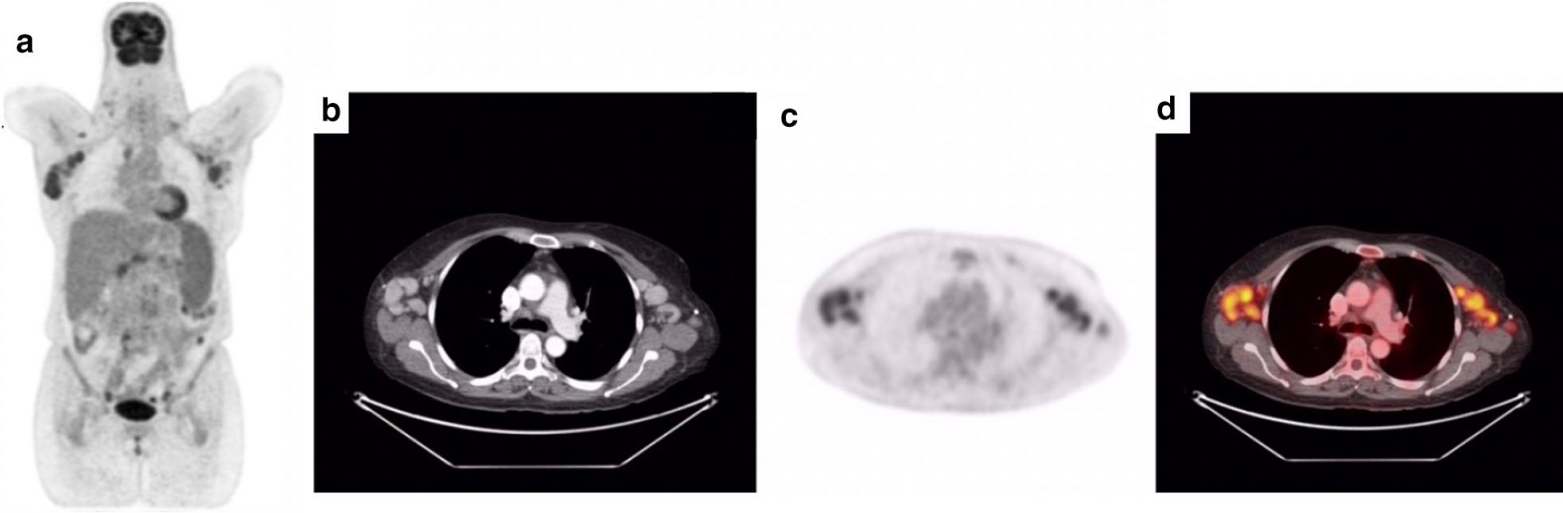
**a** displays the PET scan of a subject with multicentric Castleman disease (MCD) in the coronal view. Enlarged axillary and cervical lymph nodes and an enlarged spleen are seen in **a**, demonstrating the systemic nature of MCD. **b–d** display the CT, PET, and PET/CT cross-sectional scans of the subject’s thorax, respectively. **d** displays high uptake of lymph nodes in the right and left axillary regions as shown in **a**

**Fig. 3:**
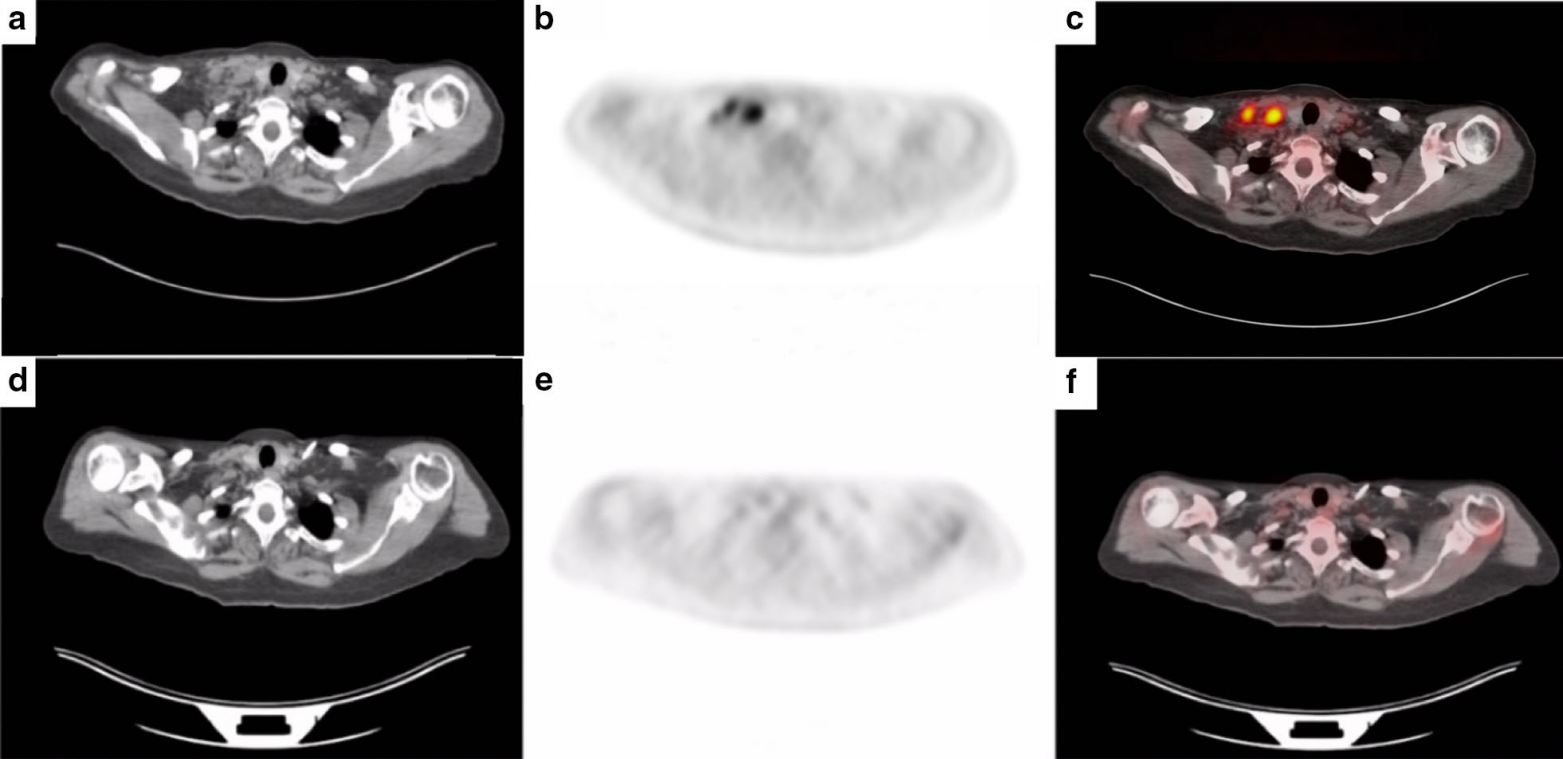
**a–c** consist of the CT, PET, and PET/CT scans, respectively, of a subject with multicentric Castleman Disease (MCD). High FDG uptake of lymph nodes can be visualized in the neck in **b** and **c**. **d–f** were taken 8 months after (**a–c**) and demonstrate decreased FDG-uptake in the same neck region, indicating that the subject’s Castleman disease treatment has been successful

Beyond identifying lymphadenopathy, FDG-PET/CT can be utilized to monitor response to treatment [39, 59]. One report on CD used FDG-PET/CT to confirm a complete anatomic and metabolic response to treatment and to enable early detection of treatment failure or relapse [60] (Figs. 2, 3).

The relapse of UCD after surgery is rare, and only a few cases have been reported [61, 62]. In contrast, HHV-8-associated MCD, POEMS-associated MCD, and iMCD frequently relapse [18, 63]. In particular, iMCD is a heterogeneous disease and not all iMCD patients respond to treatment, including anti-IL-6 therapies. The only published treatment guidelines for iMCD recommend follow-up imaging by CT or PET/CT [64]. Therefore, the role of FDG-PET/CT as both a prognostic and a monitoring tool should be further investigated.

Following rituximab therapy in HIV-positive patients with HHV-8-associated MCD, the median time to the first MCD relapse was 30 months [18]. In addition, clinicopathological features present at diagnosis have been associated with subsequent relapse [18]. In HHV-8-associated MCD, patients often have comorbid HIV infection, so the interpretation of FDG-PET/CT can be confounded by reactive changes secondary to viremia, concurrent infections, or lipodystrophy [39]. Other malignancies and inflammatory diseases may further complicate the monitoring of CD [65, 66]. Consequently, FDG-PET/CT should be interpreted together with clinical and laboratory information in the monitoring of disease activity in HHV-8-associated MCD, POEMS-associated MCD, and iMCD [67].

## Future perspectives: dual time-point (DTP) imaging and global disease assessment

Although FDG-PET/CT is effective in detecting and monitoring CD, some limitations and challenges are evident. Most significantly increased FDG uptake is not specific to CD, and several other disorders can mimic CD. As such, two nascent FDG-PET/CT techniques may prove to be useful in the assessment of CD: dual time-point FDG-PET imaging (DTP) and global disease score (GDS).

DTP involves an extra acquisition of PET data at a delayed time-point (e.g., 3 h or more after FDG administration). Most cancers demonstrate maximum FDG uptake well beyond 60 min after FDG administration, while normal tissues and inflammatory lesions generally show a decline in FDG uptake with time. Thus, DTP imaging has been demonstrated improve discrimination between cancer and inflammatory lesions [68–70]. DTP also provides additional information on disease biology [71]. Different types of malignant and inflammatory cells accumulate variable amounts of FDG due to variation in glucose-6-phosphatase. More aggressive and actively proliferating cancer cells express lower levels of glucose-6-phosphatase and exhibit rising levels of FDG uptake over a longer period, whereas the opposite is applicable in less aggressive or less proliferative cancer cells and inflammatory cells [68]. Hence, differential kinetics of FDG uptake and clearance from inflammatory and tumor cells over time may allow us to distinguish between malignancy and inflammation. Moreover, acute infectious and non-infectious inflammatory lesions behave differently from chronic lesions on delayed time-point imaging due to the different inflammatory cells involved. However, this methodology can be demanding to both implement and standardize within busy imaging departments [72]. In addition, clinician experience in interpreting morphological features on imaging is strongly related to ability to differentiate malignancy from inflammatory mimics. For these reasons, we propose that this technique be expanded to include CD. Specifically, CD tends to demonstrate a symmetric pattern in the mediastinum and hilum [9]. The performance of qualitative over quantitative metrics in inflammatory conditions has also gained some recent interest [73]. Nonetheless, a more thorough exploration of DTP is needed to assess its efficacy in differentiating between patterns of FDG uptake and clearance in active (acute) versus inactive (chronic) inflammatory lesions in CD, which could potentially help detect acute episodes of exacerbation and monitor the underlying chronic inflammatory state of CD.

**Fig. 4:**
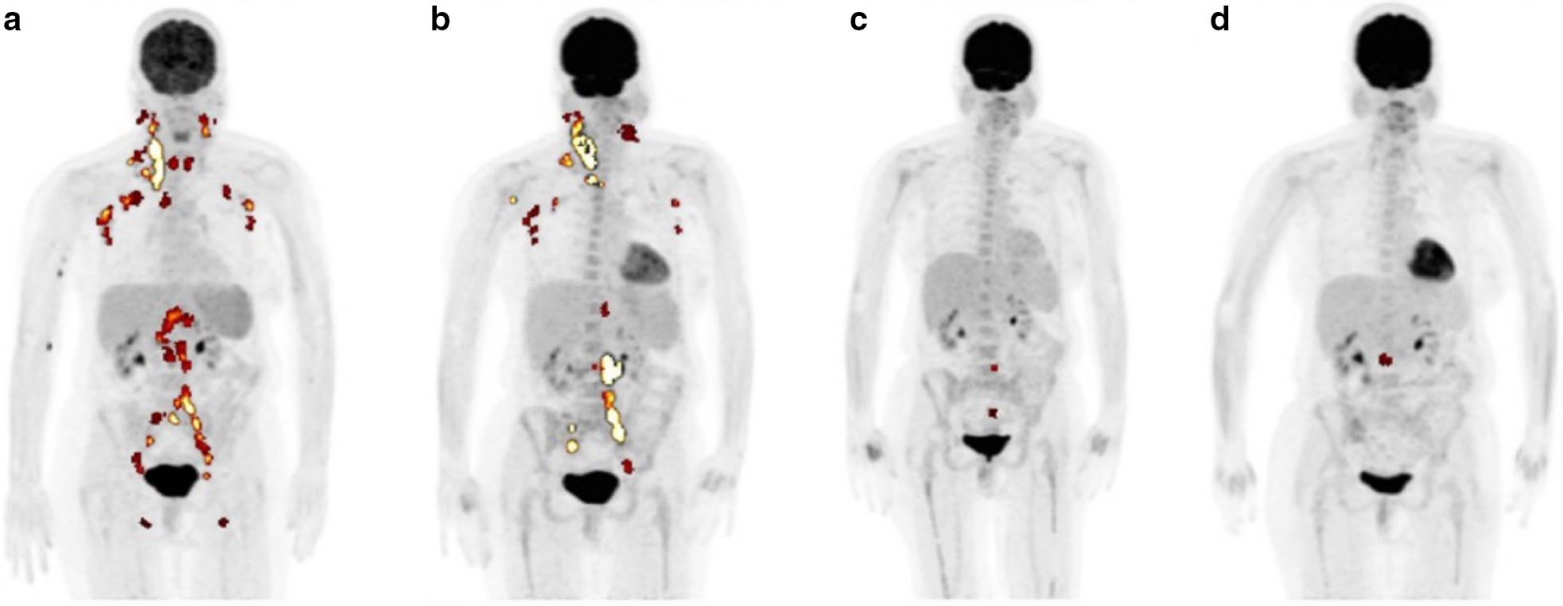
FDG-PET images of a multicentric Castleman disease (MCD) subject over 8 months. **a** and **b** demonstrate cervical, axillary, and pelvic lesions, while **c** and **d** show decrease in FDG-uptake in accordance to treatment. Total lesion glycolysis (TLG) was calculated to be 934.7, 1001.7, 17.5, and 16.4 for **a–d** respectively. TLG was calculated by multiplying the metabolic volume with FDG uptake segmented by fixed threshold methods at 41% of maximum SUV in the volume of interest (VOI) [75] by the mean standardized uptake value (SUVmean) and them summing all the intensity-volume product values from all lesions

**Fig. 5:**
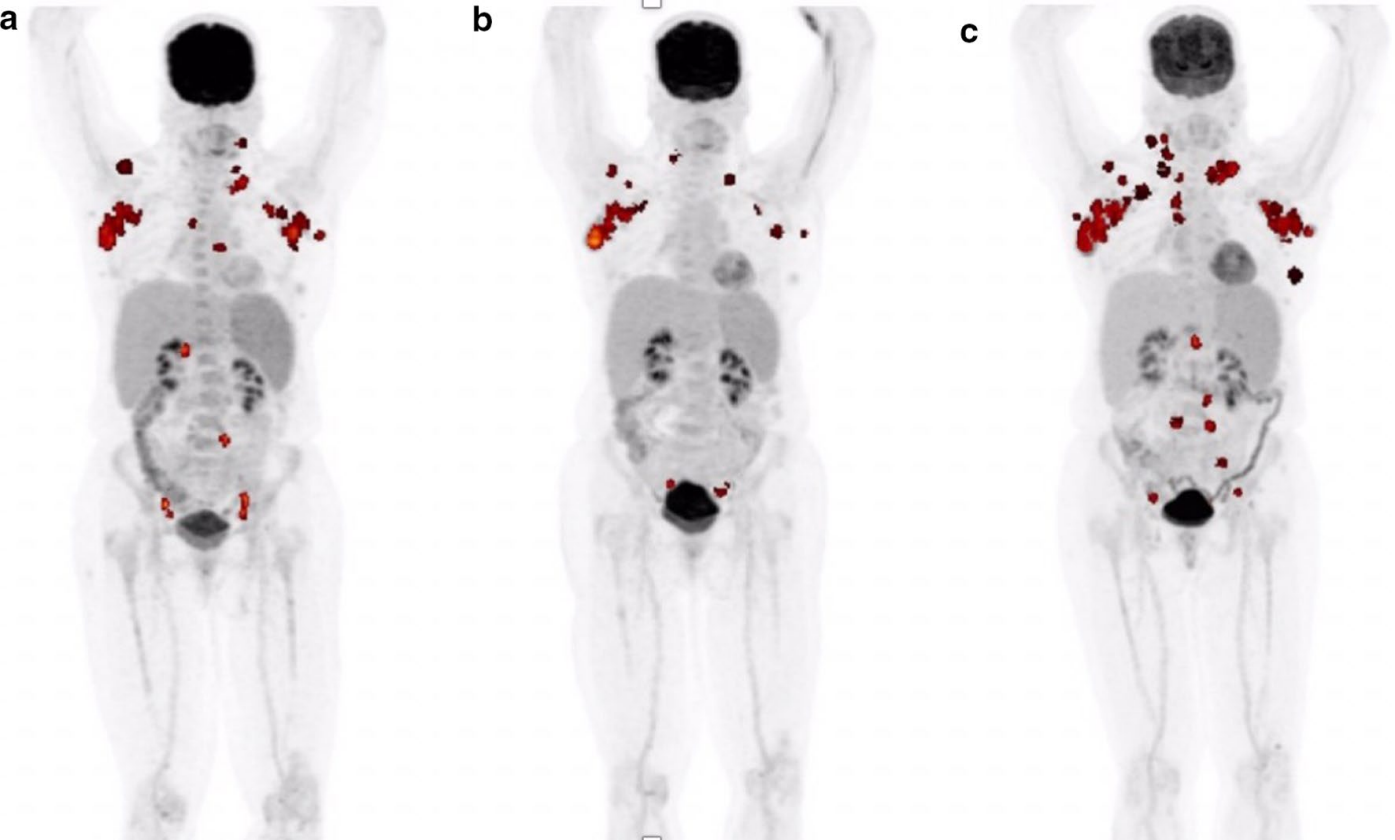
A subject with multicentric Castleman disease (MCD) with lesions in the axillary, neck, and abdomen is shown over the course of 10 months. **a** indicated the initial lesions seen in PET before treatment. **b** was taken after 4 months from the initial scan, and a decrease of lesions in the axillary, neck, and abdomen is seen. However, the disease seemed to reappear in the axillary and cervical lesions despite treatment as visualized in **c**, which was taken 6 months after the scan for **b**. Total lesion glycolysis (TLG) was calculated to be 365.9, 204.5, and 601.6 for **a–c**, respectively. TLG was calculated by multiplying the metabolic volume with FDG uptake segmented by fixed threshold methods at 41% of maximum SUV in the volume of interest (VOI) [75] by the mean standardized uptake value (SUVmean) and then summing all the intensity-volume product values from all lesions

Although CT with intravenous contrast may help with the localization of CD lesion, it cannot be used to quantify lesional activity or track it longitudinally. FDG-PET/CT imaging has been utilized in a number of other pathologies to calculate global disease burden, which uses GDS metrics including total lesion glycolysis (TLG). TLG represents a volumetric measure of FDG uptake by multiplying metabolic lesion volume (MLV) and SUVmean values obtained by using a threshold to delineate lesion activity relative to the background [74]. Single SUVmax measurements are often unreliable and unreproducible, especially when glucose uptake is heterogeneous and the disease is systemic with multiple lesions; TLG, on the other hand, is a sensitive and specific value that gives insight into the stage and progression of a disease [75–77] (Figs. 4, 5). Global disease assessment could potentially make this method of tracking disease over time of treatment easy and standardized. That is, GDS reflects the total disease burden and metabolic activity at the time of the PET examination and can be followed longitudinally to monitor disease activity and treatment efficacy [46]. This methodology has previously been applied to lymphoma patients [78–80]. Moving forward, an analogous approach should be applied toward MCD patients, especially iMCD. Despite the effectiveness and an increased use of this methodology, it has still not been widely adopted and standardized in all institutions. Nevertheless, we believe that GDS will better track the progress CD, especially given its heterogeneous presentation.

Beyond this, GDS may be used to standardize the assessment of CD progression among individuals and to monitor response to novel therapies currently under investigation [81–83]. A recent study demonstrated that IL-6-blockade refractory iMCD is responsive to sirolimus, a mammalian target of rapamycin (mTOR) inhibitor [58]. FDG-PET/CT is excellent in the early evaluation of signal transduction inhibitors like mTOR inhibitors for multiple other conditions as FDG-PET/CT detects early changes in tumor biology preceding tumor size regression, and treatment effects can be detected as early as 24 h after onset of treatment [84–87]. Therefore, FDG-PET/CT has the potential to become a powerful tool for future evaluation of biologic agents and other therapies for CD.

## Conclusion

CD is a potentially fatal disease that may overlap with numerous hematologic, inflammatory, and neoplastic diseases. FDG-PET/CT is frequently performed to visualize and localize lymph node enlargement in CD, but it has not been systematically applied in clinical practice. In the future, we believe that FDG-PET/CT and associated techniques will be useful in the diagnosis and categorization of CD, in differentiation between mimicking conditions, and monitoring of disease progression and response to treatment. Future prospective studies should be designed to assess and validate the role of this molecular imaging modality to aid in the characterization and management of this rare orphan disease.

## Data Availability

The datasets used and/or analyzed during the current study are available from the corresponding author on reasonable request.

## Abbreviations

CD: Castleman disease
DTP: Dual time-point FDG-PET imaging
FDG-PET/CT: 18F-fluorodeoxyglucose-positron emission tomography/computed tomography
MRI: Magnetic resonance imaging
GDS: Global disease score
HHV-8: Human herpesvirus-8
HIV: Human immunodeficiency virus
IgG4-RD: Immunoglobulin G4-related disease
IL: Interleukin
iMCD: Idiopathic MCD
MCD: Multicentric CD
mTOR: Mammalian target of rapamycin
MTV: Metabolic tumor volume
POEMS: Polyneuropathy, organomegaly, endocrinopathy, monoclonal gammopathy, skin changes
SUV: Standardized uptake value
SUVmax: Maximum SUV
SUVmean: Mean SUV
TLG: Total lesion glycolysis
UCD: Unicentric CD
VEGF: Vascular endothelial growth factor
